# A New Social Network Scale for Detecting Depressive Symptoms in Older Japanese Adults

**DOI:** 10.3390/ijerph17238874

**Published:** 2020-11-29

**Authors:** Seongryu Bae, Kenji Harada, Ippei Chiba, Keitaro Makino, Osamu Katayama, Sangyoon Lee, Yohei Shinkai, Hiroyuki Shimada

**Affiliations:** Department of Preventive Gerontology, Center for Gerontology and Social Science, National Center for Geriatrics and Gerontology, 7–430 Morioka, Obu, Aichi 474-8511, Japan; harada-k@ncgg.go.jp (K.H.); ichiba@ncgg.go.jp (I.C.); kmakino@ncgg.go.jp (K.M.); katayama.o@ncgg.go.jp (O.K.); sylee@ncgg.go.jp (S.L.); yshinkai@ncgg.go.jp (Y.S.); shimada@ncgg.go.jp (H.S.)

**Keywords:** social network scale, older adults, depressive symptoms, social relationships

## Abstract

Social engagement and networking deter depression among older adults. During the COVID-19 pandemic, older adults are especially at risk of isolation from face-to-face and non-face-to-face interactions. We developed the National Center for Geriatrics and Gerontology Social Network Scale (NCGG-SNS) to assess frequency of, and satisfaction with, social interactions. The NCGG-SNS consists of four domains: face-to-face/non-face-to-face interactions with family/friends. Each domain score is obtained by multiplying frequency ratings by satisfaction ratings for each item; all scores were summed to obtain a total NCGG-SNS score (range: 0–64). Additionally, face-to-face and non-face-to-face subscores were calculated. Higher scores indicated satisfactory social networking. A cohort of 2445 older Japanese adults completed the NCGG-SNS and the Geriatrics Depression Scale-Short form. Receiver Operating Characteristic (ROC) analysis and logistic regression determined predictive validity for depressive symptoms. Depressive symptoms were reported by 284 participants (11.6%). The optimal NCGG-SNS cut-off value to identify depressive symptoms was 26.5 points. In logistic regression analysis adjusted for potential confounders, lower NCGG-SNS values were significantly associated with greater prevalence of depressive symptoms. Face-to-face and non-face-to-face subscores were associated with depressive symptoms. The NCGG-SNS is a valid and useful indicator of multidimensional social networking enabling identification of depressive symptoms in older adults.

## 1. Introduction

Social isolation is highly prevalent among older adults, because of aging-related role transitions due to life events, such as retirement, death of family or friends, and limitations in physical and mental health. In addition, social networks tend to decrease in size with age. Social networks are defined as the webs of social relationships that surround individuals and the characteristics of those ties. It is a multidimensional concept that includes structural aspects of various social relationships characterized by size, density, boundedness, and homogeneity, which influence individuals’ psychosocial mechanisms such as social support, influence, engagement, or access to resources [1]. Individuals feel socially connected by interacting with others in the context of social networks and are influenced by norms and values of the networks [2]. Many studies have indicated that a lack of social networks related to mortality [3,4], disability [5], low quality of life [6], and poor mental health [7,8]. Depression is a particularly serious psychological problem among older adults [9], affecting over 322 million (4.4%) [10]. According to the fifth edition of the diagnostic and statistical manual of mental disorders (DSM-5), the diagnosis of clinical depression requires five or more symptoms during the same two-week period and at least one of the symptoms should be either depressed mood or loss of interest or pleasure [11]. The geriatric depression scale (GDS) is the most widely used screening tool for elderly depression. The cutoff score over five or six points is suggestive of depression and a score over 10 points is almost always depression [12]. Depressive symptoms can restrict older adults’ daily functional abilities and psychosocial factors such as social isolation, loneliness, and living alone in older adults [13,14,15]. A previous study found that increased risk of depressive symptoms is associated with lack of social contact [16] and poor social relationships [17]. Further, meta-analyses have indicated that interventions addressing social relationships may be effective in reducing depressive symptoms [18,19]. From this perspective, social connectivity and social relationships exhibit an inverse correlation with depression and depressive symptoms. However, most of these studies have used a broad set of theoretically representative social network domains. Determining the specific social network domains that are the most strongly related to depression and depressive symptoms will advance the basis for intervention.

The Lubben Social Network Scale (LSNS) is one of the most widely used questionnaires to quantitively assess social network size. It is used to assess social isolation in older adults by measuring the number and frequency of contact with friends and family, as well as social support received from those groups [20]. Lower LSNS scores are associated with increased depressive symptoms [21] and decreased cognitive function [22]. However, the LSNS does not assess qualitative aspects of social networks, such as satisfaction with social contacts. Teo, Choi, and Valenstein suggested that it is important to assess both quantity and quality of social relationships with respect to risk of depression [23]. However, to the best of our knowledge, no extant psychometric measures evaluate both quantity and quality of social interactions.

In addition, in the new era of the COVID-19 pandemic, it is more critical than ever to blunt the impact of social isolation and bolster social connectivity. One possible way to do so is to use information and communication technology (ICT) to help maintain contact with social networks. ICT includes communication devices or applications, such as mobile phones, computers, network hardware, and software. Social media or social networking services (SNS) are a product of the evolution of ICT and it is an online platform that people use to build social networks or social relationships with other people [24]. In Japan, the proportion of Internet and SNS users aged over 60 years has increased dramatically from 49.7% and 26.4%, respectively, in 2018 to 74.1% and 45.1%, respectively, in 2019 [25]. The utilization of ICT technology such as the Internet and social media could be a valuable tool to expand social networks and promote communication with family and friends. Using ICT technology for communication could reduce perceived social isolation and loneliness in older adults [26,27]. Thus, it is important, especially during the COVID-19 pandemic, to promote not only face-to-face interactions among older adults but also virtual interactions, such as via phone, email, and social network services, to prevent social isolation, depression, and loneliness.

Therefore, we developed a new National Center for Geriatrics and Gerontology Social Network Scale (NCGG-SNS), which assesses face-to-face and non-face-to-face interactions and considers both quantitative and qualitative aspects of social interaction. The goal of the study was to assess the predictive validity of the NCGG-SNS for depressive symptoms in older adults.

## 2. Material and Methods

### 2.1. Participants

This study involved community-dwelling older adults enrolled from a subcohort of the National Center for Geriatrics and Gerontology–Study of Geriatric Syndromes, a population-based national cohort study. This subcohort targeted community-dwelling older adults aged 60 or more, who lived in Chita City, Aichi Prefecture, Japan. An invitation letters for the baseline survey was sent to older adults (n = 9027) and 2728 of them participated from October 2019 to February 2020. We applied the following exclusion criteria: (1) history of dementia (*n* = 2), mild cognitive impairment (*n* = 1), stroke (*n* = 130), Parkinson’s disease (*n* = 10), and/or clinical depression (*n* = 59); (2) certified by the national long-term care insurance system as having a functional disability (*n* = 2); (3) lack of independence in basic activities of daily living, such as eating, bathing, grooming, walking, and stair-climbing (*n* = 9); (4) severe cognitive impairment based on Mini-Mental State Examination (MMSE) score ≤ 20 [28,29] (*n* = 31); and (5) having missing data for these criteria or any other variables included our analysis (*n* = 39). After applying exclusion criteria, data from 2445 participants were analyzed in the present study.

Participants participated in assessments at the community center. All assessments were conducted by trained nurses and study assistants. Before the commencement of the study, all staff received training from the authors regarding appropriate protocols for conducting assessments.

We obtained written informed consent from all participants prior to their inclusion in the study. The study was conducted in accordance with the Declaration of Helsinki, and the ethics committee of the National Center for Geriatrics and Gerontology approved the study protocol (Approval number: 1249-3).

### 2.2. Measures

#### 2.2.1. Depressive Symptoms

Self-report screening tools to identify depressive symptoms were deemed suitable for this community-based study. The 15-item version of the GDS has been validated to screen for depressive symptoms in older people [30,31]. Higher scores indicate more depressive symptoms. In the present study, the participants who scored ≥6 on the GDS were considered to have depressive symptoms. The cut-off score of ≥6 has sensitivity of 82% and a specificity of 75% compared with a structured clinical interview for depression [32].

#### 2.2.2. Social Network Scale

We developed a new self-report scale, the NCGG Social Network Scale (NCGG-SNS), for assessing multidimensional social networks (Appendix A). The scale assesses the following domains: (1) face-to-face interactions with family; (2) face-to-face interactions with friends; (3) non-face-to-face interactions with family using telephone, letter, or email; and (4) non-face-to-face interactions with friends using telephone, letter, or email. For each domain, participants assessed the frequency of interactions (0 = *none*, 1 = *several times a year*, 2 = *several times a month*, 3 = *several times a week*, and 4 = *every day*) and satisfaction with the interactions (only if the interactions were reported; 1 = *very dissatisfied*, 2 = *somewhat dissatisfied*, 3 = *somewhat satisfied*, and 4 = *very satisfied*). A value for each item was obtained by multiplying the frequency rating by the satisfaction rating; values were summed to obtain the total NCGG-SNS score, which ranged from 0 to 64. For secondary analysis, we also calculated the face-to-face subscore (domain 1 plus 2) and the non-face-to-face subscore (domain 3 plus 4), each of which ranged from 0 to 32. 

A panel of five experts validated the scale using the content validity index (CVI) [33,34]. Five panel members represented geriatric and health science specialists; the educational background for all the experts was a Ph.D. in health and sports science or rehabilitation science. The experts were asked to answer a questionnaire developed for the assessment of the scale by rating the clarity, concreteness, essentiality, and importance of each item using a 4-point Likert scale (e.g., 1 = not clear, 2 = not very clear, 3 = somewhat clear 4 = very clear). The CVI of each item was calculated based on the experts’ ratings. The CVI score was computed as the number of experts giving a rating of 3 or 4, divided by the total number of experts. Values range from 0 to 1 where CVI > 0.79, the item is relevant, between 0.70 and 0.79, the item needs revision, and if the value is below 0.70 the item is eliminated [34]. The mean CVI scores of the five experts in clarity, concreteness, essentiality, and importance were 0.90, 0.80, 1.00, and 0.98, respectively, indicating a high overall content validity of the scale.

#### 2.2.3. Other Covariates

The following covariates were assessed through face-to-face interviews: age, sex, comorbidities (hypertension, diabetes mellitus, heart disease, hyperlipidemia, respiratory disease, and osteoarthritis), number of medications (all drugs continuously prescribed by a doctor), educational, and living arrangement (living alone or cohabiting). We also included covariates of body mass index, current alcohol consumption (whether they drank alcohol regularly), smoking status (current, former, or never), global cognitive function, slow gait speed, and physical inactivity. Global cognitive function was measured using the MMSE. Gait speed was measured by asking participants to walk normally along a 6.4-m walkway, with data collected from a 2.4-m section in the middle of the walkway. Gait speed under 1.0 m/s was defined as a slow gait [22]. Physical inactivity was evaluated by asking the following: (1) “Do you engage in more than moderate levels of physical exercise or sports aimed at health?” and (2) “Do you engage in low levels of physical exercise aimed at health?” Participants who responded “no” to both questions were defined as being inactive [35]. 

### 2.3. Statistical Analysis

Data were analyzed using IBM SPSS Statistics 25 (IBM Japan, Tokyo, Japan). Two-tailed probability values of <0.05 were considered statistically significant. Independent sample *t*-tests were used to determine the characteristics that differed between participants with and without depressive symptoms. Categorical variables were compared using Pearson chi-square tests. Residuals followed the *t*-distribution, such that *t* > 1.96 indicated *p* < 0.05. Internal consistency of the NCGG-SNS was evaluated using Cronbach’s α coefficient. Internal consistency was considered adequate if Cronbach’s α values were >0.70 [36,37]. The test–retest reliability of each component of the NCGG-SNS was assessed by intraclass correlation coefficient (ICC) with a 95% confidence interval (CI). ICC greater than 0.90 is indicative of excellent reliability [38]. For primary analyses, we divided the NCGG-SNS into two categories according to the optimal cut-off points to identify the subjects with depressive symptoms based on receiver operating characteristic (ROC) curve analyses using the closest to (0, 1) criteria. With high NCGG-SNS values in the ROC analyses set as references, primary binomial logistic regression analysis was performed with depressive symptoms as the dependent variable and NCGG-SNS score as the independent variable. Primary analysis included the crude model and the model adjusted for covariates, namely age, sex, comorbidities (hypertension, diabetes mellitus, heart disease, hyperlipidemia, respiratory disease, and osteoarthritis), number of medications, educational, living arrangement, body mass index, current alcohol consumption, smoking status, MMSE score, slow gait speed, and physical inactivity. These confounding factors have shown associations with depression or depressive symptoms in previous studies [39,40,41,42,43]. In secondary analyses, associations between prevalence of depressive symptoms and each of face-to-face and non-face-to-face subscores were analyzed using adjusted binomial logistic regression including the same covariates as in the primary analysis. Data are presented as odds ratios (ORs) with 95% CIs.

## 3. Results

### 3.1. Participants’ Characteristics

**Figure 1:**
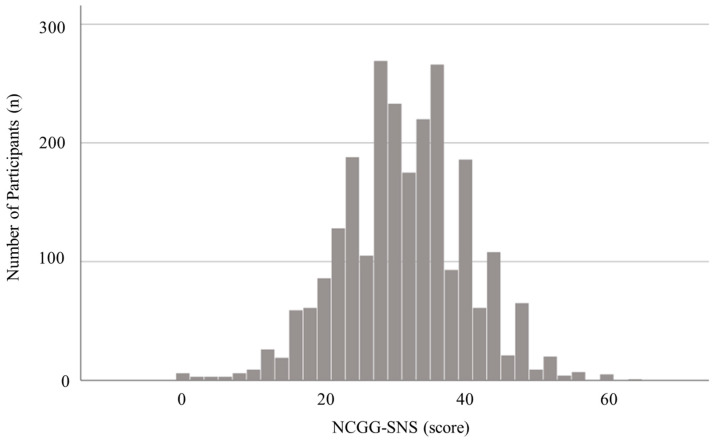
Histogram of National Center for Geriatrics and Gerontology Social Network Scale (NCGG-SNS) scores for all participants.

Table 1 presents the characteristics of the participants. Of the 2445 participants enrolled in the present study, 284 (11.6%) exhibited depressive symptoms. Differences in baseline characteristics between participants with and without depressive symptoms are shown in Table 1. There were significant differences in age (*p* = 0.011), sex (*p* = 0.007), prevalence of hypertension (*p* = 0.036), number of medications (*p* < 0.001), educational (*p* < 0.001), living arrangement (*p* < 0.001), alcohol consumption (*p* = 0.036), smoking status (*p* < 0.001), MMSE score (*p* < 0.001), proportion with slow gait (*p* < 0.001), and physical inactivity (*p* < 0.001). The average NCGG-SNS score was 30.9 ± 9.0 (Figure 1); participants with depressive symptoms exhibited significantly lower scores than those without depressive symptoms (*p* < 0.001).

### 3.2. Reliability and Validity of the NCGG-SNS

We assessed internal reliability of the NCGG-SNS using Cronbach’s α coefficient, which was 0.69 in the sample. Interitem correlations were moderate to high for the scale (item-test): the coefficients ranged from 0.30 to 0.60 (Table 2). Regarding test–retest reliability, the ICC for the total score was 0.96 (95% CI: 0.88–0.99). Thus, the test–retest reliability of the total score of the NCGG-SNS was in an acceptable range.

### 3.3. Cut-off Point of the NCGG-SNS

**Figure 2:**
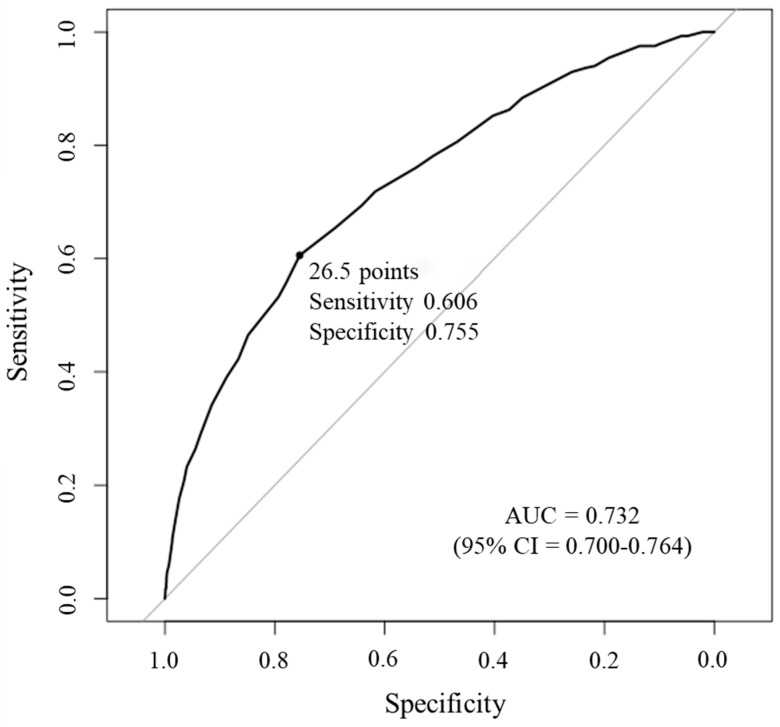
Receiver operating characteristic for NCGG-SNS used to detect the participants with depressive symptoms.

Figure 2 presents the results of ROC curve analyses of the correlation between NCGG-SNS scores and depressive symptoms. The optimal NCGG-SNS cut-off score defined by the closest to (0, 1) criteria for identifying participants with depressive symptoms was 26.5 points. This value had a sensitivity and specificity of 0.606 and 0.755, respectively. The accuracy of the NCGG-SNS for discriminating participants with depressive symptoms was 0.732, according to the area under the curve.

### 3.4. Binomial Logistic Regression Analysis

In the primary logistic regression analysis, participants who scored ≤27 on the NCGG-SNS exhibited significantly greater prevalence of depressive symptoms in both the crude model (OR: 4.72, 95% CI: 3.65–6.11, *p* < 0.001) and adjusted model (OR: 3.79, 95% CI: 2.89–4.99, *p* < 0.001). In the adjusted model, depressive symptoms were significantly associated with number of medications (OR: 1.10, 95% CI, 1.04–1.17, *p* < 0.001), living alone (OR: 1.81; 95% CI: 1.29–2.52, *p* = 0.001), regularly drinking alcohol (OR: 0.72, 95% CI: 0.54–0.98, *p* = 0.033), slow gait speed (OR: 1.86, 95% CI: 1.35–2.56, *p* < 0.001), and physical inactivity (OR: 1.57, 95% CI: 1.17–2.10, *p* = 0.002; Table 3). In secondary analyses, reduced depressive symptoms were associated with both higher NCGG-SNS face-to-face subscores (OR: 0.87, 95% CI: 0.85–0.90, *p* < 0.001) and higher non-face-to-face subscores (OR: 0.96, 95% CI: 0.93–0.99, *p* = 0.005) in a model that adjusted for the same confounders as the primary analysis.

## 4. Discussion

This study aimed to determine the predictive validity of a new instrument that measures social networking, in terms of the instrument’s ability to predict depressive symptoms. The instrument considered face-to-face and non-face-to-face interactions and both quantitative and qualitative aspects of social interaction. The internal consistency of the NCGG-SNS was reasonable, with a Cronbach’s α coefficient of 0.69, and test–retest reliability as measured by ICC was 0.96, which indicates high reliability [38]. Regarding internal consistency, a Cronbach’s α coefficient of >0.70 and an ICC greater than 0.90 is considered sufficient [36,37]. These results are similar to studies of the LSNS in various languages and populations, with values ranging from 0.83 to 0.86 [44,45].

The groups without and with depressive symptoms accounted for 2161 (88.4%) and 284 (11.6%) participants of the total study population, respectively. In a study of community-dwelling Japanese older persons, the prevalence of depressive symptoms using six as the GDS cut-off score was 10.1% [46], and another study reported a value of 15.8% [47]. Our participants demonstrated similar prevalence of depressive symptoms as have participants in previous studies. Significant differences were observed among groups with and without depressive symptoms with respect to age, sex, hypertension, education, living alone, regular alcohol consumption, smoking status, MMSE score, gait speed, physical inactivity, and NCGG-SNS score. These results are generally consistent with the results of previous studies of depressive symptoms in Japanese participants [42,47,48,49]. In addition, prior studies have shown close social networking is preventative against depression in older persons [50]. Accordingly, we found differences between groups in the NCGG-SNS scores that assessed social networks. 

The major finding of this study is that poor social interaction was associated with higher prevalence of depressive symptoms. This result suggests that frequent interactions and higher satisfaction with interactions with families, neighbors, and friends, regardless of whether such interactions are face-to-face, could decrease depressive symptoms. Furthermore, we found the newly developed NCGG-SNS was effective for detecting depressive symptoms. A previous systematic review found that access to large social networks was associated with decreased depressive symptoms among community-dwelling older adults in Asia [51]. Yang et al. examined social contact and depressive symptoms among 62,845 individuals over 65 years of age, finding the frequency of social contact with neighbors was the most powerful factor associated with older adults’ depressive symptoms [16]. Further, although depression is a significant risk factor for elder abuse, the increased level of social support and social networks are expected to have a higher protective effect [52,53]. According to a study examining relationships between social structure, social support, and depressive symptoms among older adults in the United States, there were no clear reciprocal associations between social network structure and depressive symptoms, although the number of close ties of contact was associated with later depressive symptoms [54]. They measured only the number of close ties and frequency of contact as a social network structure. However, these previous studies evaluated the frequency of social interactions not the degree of satisfaction with such interactions. Our scale evaluates not only the frequency of social interactions but also the degree of satisfaction with interactions with families, neighbors, and friends. Negative social interactions are associated with depression [7] and suicide [55]. Kabo et al. examined social networks and depressive symptoms in older African Americans and observed that the negative social relations had larger effects on depressive symptoms than positive relations. Thus, satisfactory social networks have an important role in improving depressive symptoms [56]. Thus, it is important to assess satisfaction with social networks. The mechanisms underlying the relationship between social networks and depression are not yet well understood. One possible explanation is that psychological and social factors act as a buffer against depression in the context of stressful events and biological risks [57]. Another possible explanation is that reduced isolation through improved access to social networks increases sharing of health and self-management information [50]. 

In addition, considering recent developments in communication methods, there is a need to examine the relationship between mental health and communication, including both face-to-face and non-face-to-face interactions. As a secondary analysis, we examined the associations between each of face-to-face and non-face-to-face subscores of the NCGG-SNS and prevalence of depressive symptoms. Both higher NCGG-SNS face-to-face and higher non-face-to-face subscores were significantly associated with reduced depressive symptoms. Previous studies have shown that non-face-to-face interactions may improve mental health, including depression and loneliness [26,27]. It has also been shown that face-to-face and non-face-to-face interactions may contribute to mental health in different ways [58]. Therefore, examining interactions with mental health may require treating face-to-face and non-face-to-face interactions independently and simultaneously. Our study suggests both face-to-face and non-face-to-face interactions may affect depressive symptoms independently of each other. The ongoing COVID-19 outbreak and government-imposed containment measures, including self-isolation and social distancing, have a significant impact on mental health worldwide. Older adults are experiencing greater depression and loneliness than before the pandemic [59]. The findings of Di Nicola et al. reveal that major depression and low 25- hydroxyvitamin D serum levels may predict a higher load of psychological distress in patients with mood disorders during stressful events such as the COVID-19 outbreak [60]. During the COVID-19 outbreak, digital spaces are switching from being an amenity to a necessity [61]; therefore, non-face-to-face interactions using SNSs or digital devices may be useful not only as a substitute for in-person interactions but also as a source of additional benefit for mental health.

The major strengths of the present study are its large sample size of community-dwelling older adults and the comprehensive set of assessments. The clinical practical implication of this study is that health care professionals and social service agencies need to pay attention to poor social networks in the elderly, suggesting the need to develop intervention programs to promote mental health through increasing strong social ties and enhancing the quality of social networks. However, this study has some limitations. First, we used cross-sectional data. Therefore, we were unable to determine whether there was a causal relationship between social relationships and depressive symptoms. Future studies should use longitudinal or randomized controlled trials to examine the effects of interventions on depressive symptoms. Second, our participants were relatively healthy community-dwelling older adults who were able to participate voluntarily in assessments at the community center. This might have excluded older adults with severe depressive symptoms who were likely unwilling to participate in this study. Therefore, the results of our study cannot be generalized to all older Japanese adults. Third, we did not measure psychiatric illnesses (other than depression), which may be important covariates in the relationship between depressive symptoms and social networks. We plan to examine this issue in future studies.

## 5. Conclusions

In conclusion, we developed the NCGG-SNS scale as a new social networking scale that considers face-to-face and non-face-to-face interactions and assessed the relationship between scale scores and depressive symptoms. NCGG-SNS is a valid and useful indicator of multidimensional social networking that can identify depressive symptoms in community-dwelling older adults. Further research is needed to identify the existence of any causal associations between NCGG-SNS scores and depressive symptoms.

## Figures and Tables

**Table 1:** Characteristics of all participants with and without depressive symptoms.

Variables	All Participants (*n* = 2445)	Without Depressive Symptoms (*n* = 2161)	With Depressive Symptoms (*n* = 284)	*p* Value
Age, years	72.5 (6.7)	72.4 (6.6)	73.5 (7.2)	0.011
Sex				0.007
Women, n (%)	1348 (55.1)	1213 (56.1)	135 (47.5)	
Men, n (%)	1097 (44.9)	948 (43.9)	149 (52.5)	
Hypertension, n (%)	992 (40.6)	860 (39.7)	132 (46.5)	0.036
Diabetes, n (%)	325 (13.3)	278 (12.9)	47 (16.5)	0.104
Heart disease, n (%)	371 (15.2)	323 (14.9)	48 (16.9)	0.438
Hyperlipidemia, n (%)	828 (33.9)	717 (33.2)	111 (39.1)	0.056
Respiratory disease, n (%)	339 (13.9)	301 (13.9)	38 (13.4)	0.873
Osteoarthritis, n (%)	474 (19.4)	410 (19.0)	64 (22.5)	0.178
Medications, number	2.9 (2.8)	2.3 (2.7)	3.9 (3.3)	<0.001
Educational, years	12.6 (2.5)	12.6 (2.5)	12.1 (2.4)	<0.001
Living alone, n (%)	320 (13.1)	249 (11.5)	71 (25.0)	<0.001
BMI, kg/m^2^	23.3 (3.2)	23.3 (3.1)	23.5 (3.3)	0.314
Regular alcohol consumption, n (%)	1085 (44.4)	976 (45.2)	109 (38.4)	0.036
Smoking status				<0.001
Former smoker, n (%)	793 (32.4)	684 (31.7)	109 (38.4)	
Current smoker, n (%)	198 (8.1)	168 (7.8)	30 (10.6)	
MMSE, score	27.6 (2.3)	27.7 (2.2)	27.1 (2.5)	<0.001
Slow gait speed, n (%)	405 (16.6)	318 (14.7)	87 (30.6)	<0.001
Physical inactivity, n (%)	541 (22.1)	438 (20.3)	103 (36.2)	<0.001
GDS, score	2.6 (2.5)	1.9 (1.5)	8.0 (1.9)	<0.001
NCGG-SNS, score	30.9 (9.0)	31.8 (8.6)	24.2 (9.1)	<0.001

Values are presented as mean (SD) or n (%). NCGG-SGS, National Center for Geriatrics and Gerontology-Social Network Scale; GDS, Geriatric Depression Scale; BMI, Body Mass Index; MMSE, Mini-Mental State Examination. Statistical significance was determined as *p* < 0.05.

**Table 2:** Pearson’s bivariate correlations (r) matrix, intertotal correlations, and Cronbach’s α coefficients for the NCGG-SNS ratings.

NCGG-SNS Ratings	1	2	3	4	5	6	7	ITC ^a^	A ^b^
1. Face-to-face with family, frequency								0.30 **	0.70
2. Face-to-face with friends, frequency	−0.02							0.57 **	0.66
3. Non-face-to-face with family, frequency	0.05	0.17						0.60 **	0.65
4. Non-face-to-face with friends, frequency	0.02	0.25	0.30					0.58 **	0.64
5. Face-to-face with family, satisfaction	0.36	0.03	0.06	0.05				0.48 **	0.68
6. Face-to-face with friends, rating	0.04	0.59	0.16	0.27	0.17			0.57 **	0.62
7. Non-face-to-face with family, satisfaction	0.05	0.11	0.59	0.12	0.22	0.24		0.49 **	0.65
8. Non-face-to-face with friends, rating	0.05	0.23	0.21	0.63	0.15	0.41	0.24	0.52 **	0.62

** *p* < 0.01. ^a^ intertotal correlation, ^b^ Cronbach’s α reliability coefficient.

**Table 3:** Crude and adjusted odds ratios and 95% confidence intervals for associations with depressive symptoms.

Variables	Crude Model			Adjusted Model		
	Odds Ratio	95% CI	*p* Value	Odds Ratio	95% CI	*p* Value
NCGG-SNS						
Scores ≥27	reference			reference		
Scores <27	4.72	3.65–6.11	< 0.001	3.79	2.89–4.99	< 0.001
Covariates						
Age, years				0.99	0.97–1.01	0.330
Women, yes				0.81	0.54–1.22	0.318
Hypertension, yes				0.91	0.68–1.24	0.560
Diabetes, yes				0.83	0.56–1.23	0.341
Heart disease, yes				0.74	0.50–1.09	0.124
Hyperlipidemia, yes				1.13	0.84–1.52	0.411
Respiratory disease, yes				0.78	0.53–1.15	0.206
Osteoarthritis, yes				1.24	0.89–1.74	0.209
Medications, number				1.10	1.04–1.17	<0.001
Educational, years				0.96	0.91–1.02	0.215
Living alone, yes				1.81	1.29–2.52	0.001
BMI, kg/m^2^				0.99	0.95–1.04	0.769
Regular alcohol consumption, yes				0.72	0.54–0.98	0.033
Never smoker				reference		
Former smoker				1.30	0.88–1.94	0.190
Current smoker				1.29	0.76–2.18	0.343
MMSE, score				0.96	0.90–1.02	0.186
Slow gait speed, yes				1.86	1.35–2.56	<0.001
Physical inactivity, yes				1.57	1.17–2.10	0.002

NCGG-SGS, National Center for Geriatrics and Gerontology-Social Network Scale; BMI, Body Mass Index; CI; Confidence Interval; MMSE, Mini-Mental State Examination. Statistical significance was determined as *p* < 0.05.

## Appendix A

National Center for Geriatrics and Gerontology-Social Network Scale (NCGG-SNS)

[Questions] Please answer the questions concerning your interactions with others in your daily life. Here, interaction refers to verbal or written communication with others, and does not include simple greetings.

1-a. How often do you meet and interact with your family in person?

(1) Every day; (2) Several times a week; (3) Several times a month; (4) Several times a year; (5) No interaction.

1-b. How satisfied are you when you meet and interact with your family in person?

(1) Not satisfied at all; (2) Not very satisfied; (3) Somewhat satisfied; (4) Very satisfied

2-a. How often do you meet and interact with your friends in person?

(1) Every day; (2) Several times a week; (3) Several times a month; (4) Several times a year; (5) No interaction

2-b. How satisfied are you when you meet and interact with your friends in person?

(1) Not satisfied at all; (2) Not very satisfied; (3) Somewhat satisfied; (4) Very satisfied

3-a. How often do you interact by phone, letter, email, etc., with family members whom you don’t usually meet in person?

(1) Every day; (2) Several times a week; (3) Several times a month; (4) Several times a year; (5) No interaction

3-b. How satisfied are you when you interact by phone, letter, email, etc., with family members whom you don’t usually meet in person?

(1) Not satisfied at all; (2) Not very satisfied; (3) Somewhat satisfied; (4) Very satisfied

4-a. How often do you interact by phone, letter, email, etc., with friends whom you don’t usually meet in person?

(1) Every day; (2) Several times a week; (3) Several times a month; (4) Several times a year; (5) No interaction

4-b. How satisfied are you when you interact by phone, letter, email, etc., with friends whom you don’t usually meet in person?

(1) Not satisfied at all; (2) Not very satisfied; (3) Somewhat satisfied; (4) Very satisfied

## References

[1] Berkman L.F., Glass T., Brissette I., Seeman T.E. (2000). From social integration to health: Durkheim in the new millennium. Soc. Sci. Med..

[2] Ashida S., Heaney C.A. (2008). Differential associations of social support and social connectedness with structural features of social networks and the health status of older adults. J. Aging Health.

[3] Holt-Lunstad J., Smith T.B., Layton J.B. (2010). Social relationships and mortality risk: A meta-analytic review. PLoS Med..

[4] Shye D., Mullooly J.P., Freeborn D.K., Pope C.R. (1995). Gender differences in the relationship between social network support and mortality: A longitudinal study of an elderly cohort. Soc. Sci. Med..

[5] Seeman T.E., Berkman L.F., Kohout F., Lacroix A., Glynn R., Blazer D. (1993). Intercommunity variations in the association between social ties and mortality in the elderly. A comparative analysis of three communities. Ann. Epidemiol..

[6] Becker T., Leese M., Clarkson P., Taylor R.E., Turner D., Kleckham J., Thornicroft G. (1998). Links between social network and quality of life: An epidemiologically representative study of psychotic patients in south London. Soc. Psychiatry Psychiatr. Epidemiol..

[7] Chou K.L., Liang K., Sareen J. (2011). The association between social isolation and DSM-IV mood, anxiety, and substance use disorders: Wave 2 of the National Epidemiologic Survey on Alcohol and Related Conditions. J. Clin. Psychiatry.

[8] Cohen S. (2004). Social relationships and health. Am. Psychol..

[9] Allan C.E., Valkanova V., Ebmeier K.P. (2014). Depression in older people is underdiagnosed. Practitioner.

[10] World Health Organization (2017). Depression and Other Common Mental Disorders. https://www.who.int/publications-detail/depression-global-health-estimates.

[11] American Psychiatric Association (2013). American Psychiatric Association DSM-5 Task Force. Diagnostic and Statistical Manual of Mental Disorders: DSM-5.

[12] Wancata J., Alexandrowicz R., Marquart B., Weiss M., Friedrich F. (2006). The criterion validity of the Geriatric Depression Scale: A systematic review. Acta Psychiatr. Scand..

[13] Gu L., Yu M., Xu D., Wang Q., Wang W. (2020). Depression in Community-Dwelling Older Adults Living Alone in China: Association of Social Support Network and Functional Ability. Res. Gerontol. Nurs..

[14] Hsueh Y.C., Chen C.Y., Hsiao Y.C., Lin C.C. (2019). A longitudinal, cross-lagged panel analysis of loneliness and depression among community-based older adults. J. Elder Abus. Negl..

[15] Vanoh D., Shahar S., Yahya H.M., Hamid T.A. (2016). Prevalence and Determinants of Depressive Disorders among Community-dwelling Older Adults: Findings from the Towards Useful Aging Study. Int. J. Gerontol..

[16] Yang J., Park E.C., Lee S.A., Lee J.E., Choi D.W., Chae W., Jang S.I. (2018). The Association between Social Contacts and Depressive Symptoms among Elderly Koreans. Psychiatry Investig..

[17] Barger S.D., Messerli-Burgy N., Barth J. (2014). Social relationship correlates of major depressive disorder and depressive symptoms in Switzerland: Nationally representative cross sectional study. BMC Public Health.

[18] Barbato A., D’Avanzo B. (2008). Efficacy of couple therapy as a treatment for depression: A meta-analysis. Psychiatr. Q..

[19] Pfeiffer P.N., Heisler M., Piette J.D., Rogers M.A., Valenstein M. (2011). Efficacy of peer support interventions for depression: A meta-analysis. Gen. Hosp. Psychiatry.

[20] Lubben J., Blozik E., Gillmann G., Iliffe S., von Renteln Kruse W., Beck J.C., Stuck A.E. (2006). Performance of an abbreviated version of the Lubben Social Network Scale among three European community-dwelling older adult populations. Gerontologist.

[21] Sakurai R., Kawai H., Suzuki H., Kim H., Watanabe Y., Hirano H., Ihara K., Obuchi S., Fujiwara Y. (2019). Poor Social Network, Not Living Alone, Is Associated With Incidence of Adverse Health Outcomes in Older Adults. J. Am. Med. Dir. Assoc..

[22] Rohr S., Lobner M., Guhne U., Heser K., Kleineidam L., Pentzek M., Fuchs A., Eisele M., Kaduszkiewicz H., Konig H.H. (2020). Changes in Social Network Size Are Associated With Cognitive Changes in the Oldest-Old. Front. Psychiatry.

[23] Teo A.R., Choi H., Valenstein M. (2013). Social relationships and depression: Ten-year follow-up from a nationally representative study. PLoS ONE.

[24] Khan M.S., Khan I., Din S., Ismail H.M., Khattak R., Jan R. (2015). The impacts of ICT on the students’ Performance: A Review of Access to Information. Res. Humanit. Soc. Sci..

[25] Ministry of Internal Affairs and Communications “Communications Usage Trend Survey,” Japan, 2019. https://www.soumu.go.jp/menu_news/s-news/01tsushin02_02000148.html.

[26] Chen Y.R., Schulz P.J. (2016). The Effect of Information Communication Technology Interventions on Reducing Social Isolation in the Elderly: A Systematic Review. J. Med. Internet Res..

[27] Cotten S.R., Anderson W.A., McCullough B.M. (2013). Impact of internet use on loneliness and contact with others among older adults: Cross-sectional analysis. J. Med. Internet. Res..

[28] Folstein M.F., Folstein S.E., McHugh P.R. (1975). “Mini-mental state”. A practical method for grading the cognitive state of patients for the clinician. J. Psychiatr. Res..

[29] Tsutsumimoto K., Doi T., Makizako H., Hotta R., Nakakubo S., Kim M., Kurita S., Suzuki T., Shimada H. (2018). Social Frailty Has a Stronger Impact on the Onset of Depressive Symptoms than Physical Frailty or Cognitive Impairment: A 4-Year Follow-up Longitudinal Cohort Study. J. Am. Med. Dir. Assoc..

[30] Lesher E.L., Berryhill J.S. (1994). Validation of the Geriatric Depression Scale--Short Form among inpatients. J. Clin. Psychol..

[31] Yesavage J.A. (1988). Geriatric Depression Scale. Psychopharmacol. Bull..

[32] Friedman B., Heisel M.J., Delavan R.L. (2005). Psychometric properties of the 15-item geriatric depression scale in functionally impaired, cognitively intact, community-dwelling elderly primary care patients. J. Am. Geriatr. Soc..

[33] Hyrkas K., Appelqvist-Schmidlechner K., Oksa L. (2003). Validating an instrument for clinical supervision using an expert panel. Int. J. Nurs. Stud..

[34] Zamanzadeh V., Ghahramanian A., Rassouli M., Abbaszadeh A., Alavi-Majd H., Nikanfar A.R. (2015). Design and Implementation Content Validity Study: Development of an instrument for measuring Patient-Centered Communication. J. Caring Sci..

[35] Doi T., Shimada H., Makizako H., Tsutsumimoto K., Hotta R., Nakakubo S., Suzuki T. (2015). Mild Cognitive Impairment, Slow Gait, and Risk of Disability: A Prospective Study. J. Am. Med. Dir. Assoc..

[36] Nunnally J.C., Bernstein I.H. (1994). Psychometric Theory.

[37] Terwee C.B., Bot S.D., de Boer M.R., van der Windt D.A., Knol D.L., Dekker J., Bouter L.M., de Vet H.C. (2007). Quality criteria were proposed for measurement properties of health status questionnaires. J. Clin. Epidemiol..

[38] Koo T.K., Li M.Y. (2016). A Guideline of Selecting and Reporting Intraclass Correlation Coefficients for Reliability Research. J. Chiropr. Med..

[39] Bishwajit G., O’Leary D.P., Ghosh S., Yaya S., Shangfeng T., Feng Z. (2017). Physical inactivity and self-reported depression among middle- and older-aged population in South Asia: World health survey. BMC Geriatr..

[40] Holzel L., Harter M., Reese C., Kriston L. (2011). Risk factors for chronic depression--a systematic review. J. Affect. Disord..

[41] Kim J., Noh J.W., Park J., Kwon Y.D. (2014). Body mass index and depressive symptoms in older adults: A cross-lagged panel analysis. PLoS ONE.

[42] Shimada H., Lee S., Bae S., Hotta R. (2020). A New Life Satisfaction Scale Predicts Depressive Symptoms in a National Cohort of Older Japanese Adults. Front. Psychiatry.

[43] Tsutsumimoto K., Doi T., Shimada H., Makizako H., Hotta R., Nakakubo S., Suzuki T. (2016). Combined Effect of Slow Gait Speed and Depressive Symptoms on Incident Disability in Older Adults. J. Am. Med. Dir. Assoc..

[44] Chang Q., Sha F., Chan C.H., Yip P.S.F. (2018). Validation of an abbreviated version of the Lubben Social Network Scale (“LSNS-6”) and its associations with suicidality among older adults in China. PLoS ONE.

[45] Myagmarjav S., Burnette D., Goeddeke F. (2019). Comparison of the 18-item and 6-item Lubben Social Network Scales with community-dwelling older adults in Mongolia. PLoS ONE.

[46] Ishii S., Chang C., Tanaka T., Kuroda A., Tsuji T., Akishita M., Iijima K. (2016). The Association between Sarcopenic Obesity and Depressive Symptoms in Older Japanese Adults. PLoS ONE.

[47] Maruta M., Makizako H., Ikeda Y., Miyata H., Nakamura A., Han G., Shimokihara S., Tokuda K., Kubozono T., Ohishi M. (2020). Associations between Depressive Symptoms and Satisfaction with Meaningful Activities in Community-Dwelling Japanese Older Adults. J. Clin. Med..

[48] Adachi Y., Toyoshima K., Nishimoto R., Ueno S., Tanaka T., Imaizumi A., Arashida N., Nakamura M., Abe Y., Hakamada T. (2019). Association between plasma alpha-aminobutyric acid and depressive symptoms in older community-dwelling adults in Japan. Geriatr. Gerontol. Int..

[49] Tsujiguchi H., Thi Thu Nguyen T., Goto D., Miyagi S., Kambayashi Y., Hara A., Yamada Y., Nakamura H., Shimizu Y., Hori D. (2019). Relationship between the Intake of n-3 Polyunsaturated Fatty Acids and Depressive Symptoms in Elderly Japanese People: Differences According to Sex and Weight Status. Nutrients.

[50] Fiske A., Wetherell J.L., Gatz M. (2009). Depression in older adults. Annu. Rev. Clin. Psychol..

[51] Tengku Mohd T.A.M., Yunus R.M., Hairi F., Hairi N.N., Choo W.Y. (2019). Social support and depression among community dwelling older adults in Asia: A systematic review. BMJ Open.

[52] Dong X., Beck T., Simon M.A. (2010). The associations of gender, depression and elder mistreatment in a community-dwelling Chinese population: The modifying effect of social support. Arch. Gerontol. Geriatr..

[53] Dong X., Chen R., Chang E.S., Simon M. (2013). Elder abuse and psychological well-being: A systematic review and implications for research and policy—A mini review. Gerontology.

[54] Bui B.K.H. (2020). The relationship between social network characteristics and depressive symptoms among older adults in the United States: Differentiating between network structure and network function. Psychogeriatrics.

[55] Holma K.M., Melartin T.K., Haukka J., Holma I.A., Sokero T.P., Isometsa E.T. (2010). Incidence and predictors of suicide attempts in DSM-IV major depressive disorder: A five-year prospective study. Am. J. Psychiatry.

[56] Kabo F.W., Antonucci T.C., Jackson J.S. (2019). A Social Relations and Networks Perspective of Depressive Symptoms in Older African Americans Relative to Two Other Ethno-racial Groups. Innov. Aging.

[57] Hendrie H.C., Albert M.S., Butters M.A., Gao S., Knopman D.S., Launer L.J., Yaffe K., Cuthbert B.N., Edwards E., Wagster M.V. (2006). The NIH Cognitive and Emotional Health Project. Report of the Critical Evaluation Study Committee. Alzheimers Dement..

[58] Chan M., Li X. (2020). Smartphones and psychological well-being in China: Examining direct and indirect relationships through social support and relationship satisfaction. Telemat. Inform..

[59] Krendl A.C., Perry B.L. (2020). The impact of sheltering-in-place during the COVID-19 pandemic on older adults’ social and mental well-being. J. Gerontol. B Psychol. Sci. Soc. Sci..

[60] Di Nicola M., Dattoli L., Moccia L., Pepe M., Janiri D., Fiorillo A., Janiri L., Sani G. (2020). Serum 25-hydroxyvitamin D levels and psychological distress symptoms in patients with affective disorders during the COVID-19 pandemic. Psychoneuroendocrinology.

[61] Beaunoyer E., Dupere S., Guitton M.J. (2020). COVID-19 and digital inequalities: Reciprocal impacts and mitigation strategies. Comput. Hum. Behav..

